# Internal sphincterotomy reduces postoperative pain after Milligan Morgan haemorrhoidectomy

**DOI:** 10.1186/1471-2482-9-16

**Published:** 2009-10-24

**Authors:** Giuseppe Diana, Giovanni Guercio, Bianca Cudia, Calogero Ricotta

**Affiliations:** 1 Università degli Studi di Palermo- Dipartimento di Chirurgia Generale, d'Urgenza e dei Trapianti d'Organo- Via Liborio Giuffrè n° 5- 90100- Palermo- Italy

## Abstract

**Background:**

Over the last few years, there has been increasing attention on surgical procedures to treat haemorrhoids. The Milligan-Morgan haemorrhoidectomy is still one of the most popular surgical treatments of haemorrhoids. The aim of the present work is to assess postoperative pain, together with other early and late complications, after Milligan-Morgan haemorrhoidectomy as we could observe in our experience before and after performing an internal sphincterotomy.

**Methods:**

from January 1980 to May 2007, we operated 850 patients, but only 699 patients (median age 53) were included in the present study because they satisfied our inclusion criteria. The patients were divided into two groups: all the patients operated on before 1995 (group A); all the patients operated on after 1995 (group B). Since 1995 an internal sphincterotomy of about 1 cm has been performed at the end of the procedure. The data concerning the complications of these two groups were compared. All the patients received a check-up at one and six months after operation and a telephone questionnaire three years after operation to evalue medium and long term results.

**Results:**

after one month 507 patients (72.5%) did not have any postoperative complication. Only 192 patients (27.46%) out of 699 presented postoperative complication and the most frequent one (23.03%) was pain. The number of patients who suffered from postoperative pain decreased significantly when performing internal sphincterotomy, going from 28.8% down to 10.45% (χ^2^: 10,880; p = 0,0001); 95% Confidence Interval (CI) 24.7 to 28.9 (group A) and 10.17 to 10.72 (group B). In 51 cases (7.29%) urinary retention was registered. Six cases of bleeding (0.85%) were registered. Medium and long term follow up did not show any difference among the two groups.

**Conclusion:**

internal sphincterotomy: reduces significantly pain only in the first postoperative period, but not in the medium-long term follow up; does not increase the incidence of continence impairment when performed; does not influence the incidence of the other postoperative complications especially as regard medium and long term results.

## Background

Over the last few years, there has been increasing attention on surgical procedures to treat haemorrhoids. Several comparative studies have been performed to evaluate the procedures already available to treat second, third, and fourth-degree haemorrhoids, and new surgical techniques, such as, for instance, haemorrhoidectomy with Harmonic scalpel^® ^[1-3] and Ligasure™[4], doppler guided haemorrhoidal plexus ligation[5,6] and the stapled haemorrhoidopexy [7-13].

The most recent publications provide data on the efficacy, the results and complications from medium and long-term studies carried out on ample case series [14-20]. Despite the support offered by different authors, none of these techniques proved to reduce pain at such levels to be unanimously chosen [11].

Today, however, the Milligan-Morgan haemorrhoidectomy is still one of the most popular surgical treatments of haemorrhoids

The aim of the present work is to assess postoperative pain, together with other early and late complications, after Milligan-Morgan haemorrhoidectomy as we could observe in our experience before and after performing an internal sphincterotomy.

## Methods

Since January 1980, patients undergoing haemorrhoid treatment in our General Surgery unit have been involved in a research study on post-haemorrhoidectomy complications. Until May 2007, there were 850 operated patients in total. However, 151 patients were not included in the study because of the reasons listed in table 1.

**Table 1:** Patients excluded, because they did not fit the inclusion criteria or they had additional proctologic diseases.

Hemorrhoidal recurrence	57
Hemorrhoids and other anal/rectal diseases	67

Anal fissure	51

Polipoid lesions	9

Perianal fistula	4

Perianal abscess	3

Stapled hemorrhoidopexy	27

The operated patients available for the present study are 699, median age 53 (range 16-78) (Table 2).

**Table 2:** Distribution of haemorrhoids degree in the study group.

	**II grade**	**III grade**	**IV grade**	**tot**
Men	7	309	137	443
Women	9	155	82	256

Total	16	464	219	699

The patients underwent surgical treatment when they had at least one of the symptoms listed in table 3.

**Table 3:** Inclusion criteria of surgical treatment

Recurrent bleeding that may cause anaemia
Phlogistic or infective disorders (phlebitis, thrombophlebitis) recurring more than twice a year and forcing patients to stop their normal occupations

Mucous prolapse

Continuous or recurrent pruritus, to such an extent that it would make it socially unpleasant or cause lesions due to scratching

Sense of heaviness, of incomplete evacuation and tenesmus

In 31 cases none of the mentioned symptoms and complications were present, but the patients insisted to undergo surgery.

Before the procedure, all patients underwent proctoscopy.

The operations were carried out under general anaesthesia and infiltration of pudendal nerves with 15 ml bupivacaine with 1:200000 adrenaline. Further 5 ml of the same solution were used to dissect the haemorrhoidal nodules from the internal sphincter. Except 27 patients, who had a considerable rectal mucosal prolapse and were treated with stapled haemorrhoidopexy, all the patients underwent the same procedure, i.e. the Milligan-Morgan haemorrhoidectomy

We removed the haemorrhoidal nodule by performing an upside-down V-shaped incision on the anal dermis, without widening the surgical wound while approaching the sphincters. This was done in order to maintain ample mucous membrane bridges. Possible secondary nodules were removed trough submucosa. Furthermore we performed these technical arrangements:

• Park's suspensor ligament is not removed with the haemorrhoidal nodule (so as not to include the ligament in the transfixed stitch);

• coagulation with electrotome on the anal sphincters is avoided;

• the edges of the residual surgical wound have to be as sharp as possible.

Since 1995 an internal sphincterotomy of about 1 cm was performed at the end of the procedure, as was the case for 220 patients. We chose permanent surgical sphincterotomy, instead of temporary medical one, because at that time it was the only effective procedure in reducing postoperative sphincter spasm.

The patients were divided into two groups: patients operated on before (group A) and after 1995 (group B). The data concerning the complications of this group of patients (group B) were compared with those regarding the patients operated on before sphincterotomy (group A). No antibiotic prophylaxis has ever been administered.

On the basis of literature data and our clinical experience, it was possible to register the early endpoints (table 4).

**Table 4:** Item of follow up registered in the postoperative period.

**Early complications**	**Late complications**
Pain (VAS > 6)	Slow wound healing
Urinary retention	Incontinence
Bleeding	Anal stenosis
Edema (> 2 days)	Anal fissure
Infection	
Fever (> 5 days)	

When discharged, the patients were invited to have a check-up after one and six months from the operation, so as to assess our endpoints (table 5).

**Table 5:** Results of the study showing primary (pain) and secondary endpoints.

	**Before 1995**	**After 1995**
	**At the discharge**	
**Pts controlled after operation**	479 (%)	220 (%)
**Pain**	138 (28,81)*	23 (10,45)*
**Urinary retention**	44 (9,19)	7 (3,18)
**Bleeding**	4 (0,83)	2 (0,9)
**Edema**	33 (6,89)	7 (3,18)
**Infection**	4 (0,83)	2 (0,9)
**Fever (>5 GG.)**	15 (3,13)	4 (1,81)
		
	**After one month**	
	477 (%)	220 (%)
**Slow wound healing**	150 (31.44)	71 (32.27)
**Incontinence**	9 (1.88)	4 (1.81)
**Stenosis**	4 (0.83)	1 (0.45)
		
	**After six months**	
	477 (%)	217 (%)
**Anal fissure**	19 (3.98)	4 (1,84)
**Stenosis**	2 (0.42)	1 (0.46)
**Incontinence**	6 (1.26)	3 (1.38)
		
	**After three years**	
	322 (%)	210 (%)
**Recurrence**	7 (2.17)	6 (2.86)
**Bleeding**	22 (6.83)	25 (11.9)
**Skin tags**	13 (4.04)	15 (7.14)

*(χ^2 ^: 10,880; p = 0,0001); 95% CI 24.7 to 28.9 (group A) and 10.17 to 10.72 (group B)

In particular, we evalued pain using the visual analog scale (VAS). We considered pain as severe when: VAS > 5; patients needed administration of ketorolac three times or more a day; pain did not disappear after the third postoperative day.

Three years after the operation the patients were contacted again by phone to check the recurrence of haemorrhoids and/or other disorders and, where possible, to have a further check-up.

The study received the approval from the University School of Medicine of Palermo Ethics Committee and was in compliance with the Helsinki Declaration.

## Results

Of all 699 patients who underwent haemorrhoidectomy, 507 patients (72.5%) did not have any postoperative complication. Table 5 shows the postoperative data concerning 192 patients (27.46%) out of 699.

Pain stands as the most frequent complication (23.03%). These patients had to be administered ketorolac more than three times a day. However, pain tended to disappear spontaneously between the third and the fifth postoperative day. In 19 cases (2.7%) pain persisted for more than ten days and was accompanied by the onset of an anal fissure. The number of patients who suffered from postoperative pain decreased significantly when performing internal sphincterotomy, going from 28.8% down to 10.45% (χ^2 ^: 10,880; p = 0,0001); 95% Confidence Interval (CI) 24.7 to 28.9 (group A) and 10.17 to 10.72 (group B). We did not register any clinical impairment of continence in the two groups of patients.

In 51 cases (7.29%) urinary retention was registered (in 37 cases this complication was associated with pain or oedema or fever). The problem was solved by applying a catheter. The sphincterotomy did not reduce such complication significantly.

Six cases of bleeding (0.85%) were registered: four early cases, between the first and the second postoperative day, and two late cases. Despite the various efforts made, haemostasis was achieved by performing a transfixed stitch. The two cases of late haemorrhage showed more serious consequences. One patient had a serious anaemia with haemoglobinemia levels of 7.5 g/dl. The other patient, on the tenth day, did not show serious anaemia thanks to a further early procedure. However, he had to be transferred to intensive care, due to the onset of aspiration pneumonia.

One month after the operation 697 patients had a check-up: in 221 of these patients (31.61%) healing had not been achieved yet, whereas 5 cases (0.71%) had already been affected by stenosis. Finally, 13 patients (1.87%) complained that they sometimes were not able to control gas (8 cases) or also liquid faeces. There was not any statistically difference as regard the impairment of continence in the two groups of patients (table 4).

Six months after the operation 694 patients were evalued: 477 had the operation before 1995, whereas the remaining 217 patients had the operation after 1995. Twenty-three patients (3.31%) were diagnosed as having a fissure on one of the haemorrhoidectomy beds. The internal sphincterotomy did not reduce significantly this complication, which affected 2.59% of cases. It is worth emphasising that 21 patients out of the 23 patients who were affected by a fissure had had pain as an immediate postoperative complication. Three other patients were affected by stenosis, whereas patients suffering from incontinence decreased to nine (1.30%) and only occasionally had gas incontinence.

After three years, the results were studied on 532 patients out of the 699 patients who had been operated and answered the questionnaire. Thirteen patients (2.45%) had a recurrence and 47 patients (8.83%) reported that they occasionally experienced bleeding, though there was no evidence of haemorrhoidal recurrence. In 38 patients skin tags were found.

## Discussion

Drawing on our experience, we evalued the complications arising after surgical procedures to treat haemorrhoids and in particular we wanted to assess postoperative pain and other complications.

Among the complications, the most frequent one is surely the onset of severe pain, whose incidence in our data is in line with literature data [10-21] including studies on the use of a stapling device.

In this respect, it is worth considering the importance of performing anal divulsion/internal sphincterotomy to prevent postoperative pain, as already highlighted in several multi-centre clinical trials [22,23]. Some authors, instead, assess that the internal sphincterotomy does not influence the incidence of postoperative pain [24,25]. Sometimes pain may persist over time after cicatrisation of wounds. In most of these cases, the cause should be attributed to the formation of an anal fissure on the groove of one of the excised peduncles.

Continence-related disorders include the possible onset of various degrees of incontinence or of costiveness. Incontinence occurring after a procedure of low ligature is rare and transitory, as it resolves by itself within 3-5 days after the operation. Moreover, it is usually not above grade C Browning-Parks[26].

In our experience, the incidence of incontinence was lower than the levels reported by Graviè et al. [17] at check-up after one month (1.86% vs. 8.8%) and after six months (1.29% vs. 8.8%). Incontinence is more frequent after a Whitehead procedure, and it is also for this reason that this technique is not popular and it is indicated for selected patients under the supervision of an expert surgeon [27].

In reviewing 400 patients treated by Montorsi [28], the same author found a proportion of anal fissures amounting to 5%. In such cases, the treatment consists in regularizing the alvus and, in particularly resistant cases, performing an anal divulsion or an internal sphincterotomy.

The cases of urinary retention observed in our study (7.30%) are less than those indicated by Toyonaga T. et al. [29,30] (21.9%), and they are near the data provided by Chik B. et al. [31] (7.77%) in a study on stapled haemorroidopexy. This complication affects more male subjects, mostly aged between 40 and 60 years.

Postoperative bleeding is a particularly important complication in treating haemorroids due to its frequency, which vary between 0.6% and 10% [31,32] depending on the study considered. Sometimes bleeding may be alarming, because it may cause anaemia very rapidly in patients.

The causes of such bleeding are not easily explained: in some cases it should be attributed to falling off of an scar due to electrocoagulation, whereas in other cases it is due to the lack of a thrombus, its expulsion or its dissolution, concomitant with the falling or reabsorption of the transfixed stitch.

Haemorrhoidal recurrence stands at around 2-8% [17]. On the other hand, in the past the proportion of recurrence used to be around 14%, as already pointed out by Goligher [33]. However, one system to prevent this from occurring is to perform subcutaneous removal of these nodules.

Long-term outcomes of haemorrhoidectomy procedures include the onset of malformations of the anal canal, with varying degrees of seriousness up to cases of stenosis. In our experience, the incidence of anal stenosis is very low (0.43%). This is probably due to the fact that we avoid widening the residual surgical wound.

As regards long term recurrence (three years), in our data we found an incidence of 2.45%, which is in line with literature data and lower than methods that are considered more efficacious, such as, for instance, stapled haemorroidopexy, which is reported to have a higher incidence[34].

## Conclusion

The types and frequency of immediate, medium and long-term complications we could observe following a Milligan-Morgan haemorrhoidectomy are in line or, for some variables, even less than those described in the literature.

The present paper shows how, according to our experience, the internal sphincterotomy: reduces significantly pain only in the first postoperative period, but not in the medium-long term follow up; does not increase the incidence of continence impairment when performed; does not influence the incidence of the other postoperative complications especially as regard medium and long term results.

Our study highlights how results can be improved by implementing some technical arrangements, which expert proctologists are able to carry out. This way, in terms of results and complications, the Milligan-Morgan haemorrhoidectomy is similar to other techniques, which are recently become popular because of the lower degree of pain referred by their advertising.

## Competing interests

The authors declare that they have no competing interests.

## Authors' contributions

GD conceived of the study, participated in the coordination of the study and operated on the patients of the study. GG participated in the design, performed the statistical analysis and operated on the patients. BC helped in the statistical analysis and helped in the draft of the manuscript. CR participated in the collection of the data and in the draft of the manuscript. All authors read and approved the final manuscript.

## Authors' informations

GD: Full Professor of Surgery at the School of Medicine of the University of Palermo, Director of the Unit of General and Geriatric Surgery at the University Hospital Policlinico "P.Giaccone".

GG: researcher at the School of Medicine of the University of Palermo, resident surgeon at the Department of General, Urgency and Transplant Surgery.

BC: researcher at the School of Medicine of the University of Palermo.

CR: fellow in Digestive and Endoscopic Surgery at the School of Medicine of the University of Palermo.

## Pre-publication history

The pre-publication history for this paper can be accessed here:


